# Fractal Analysis of Brain Activity During Risky Drinking in Adolescents and Young Adults

**DOI:** 10.3390/brainsci15121256

**Published:** 2025-11-22

**Authors:** Derek Madden, Robert G. Lyday, Mohsen Bahrami, Heather M. Shappell, Jonathan H. Burdette, Paul J. Laurienti

**Affiliations:** 1 Virginia Tech-Wake Forest University School of Biomedical Engineering and Sciences, Wake Forest University School of Medicine, Winston-Salem, NC 27157, USA; derek.madden@wfusm.edu; 2 Department of Radiology, Wake Forest University School of Medicine, Winston-Salem, NC 27157, USA; robert.lyday@advocatehealth.org (R.G.L.); mohsen.bahrami@advocatehealth.org (M.B.); jonathan.burdette@wfusm.edu (J.H.B.); 3 Department of Biostatistics and Data Science, Wake Forest University School of Medicine, Winston-Salem, NC 27157, USA; heather.shappell@wfusm.edu

**Keywords:** fMRI, fractal, alcohol use disorder, regional homogeneity, amplitude of low frequency fluctuations

## Abstract

**Background/Objectives**: Despite widespread negative effects on physical and societal well-being, the neurological effects and risk factors of alcohol misuse are far from being fully understood. To broaden knowledge about inherent differences and possible changes in the brain reflecting alcohol use, we investigated functional Magnetic Resonance Imaging data in a group of young adult and adolescent individuals with varying levels of alcohol consumption from the National Consortium on Alcohol and Neurodevelopment in Adolescence dataset. **Methods**: We evaluated fractal complexity, or long-term self-memory of brain activity, using the Hurst Exponent, spontaneous neural activity using Amplitude of Low Frequency Fluctuations, and local coherence/synchronization using Regional Homogeneity. Regional values for these measures of interest were compared between risky drinkers and light drinkers, as well as between the same groups of individuals before development of any risky drinking habits. **Conclusions**: Significant differences (Cohen’s d > 0.557) in the varying measures were identified between risky and light drinkers that may point to abnormal activity patterns in regions including the insula, precuneus, and inferior frontal lobe. Importantly, a control comparison between the same groups of individuals at younger, non/light drinking ages revealed distinct differences in brain patterns, potentially consistent with the interpretation that differences in brain activity patterns among the older groups are a result of drinking patterns rather than a cause. In contrast, the differences identified in the younger groups may be potential risk factors indicating increased likelihood of engaging in heavier drinking habits.

## 1. Introduction

One of the most common neuropsychiatric disorders in the world, alcohol use disorder (AUD) is characterized by the continued, consistent consumption of alcoholic beverages despite harmful effects on mental, physical, and/or social well-being [1,2]. In the United States, nearly 30 million suffer from AUD, while an estimated 400 million live with AUD worldwide [3,4]. The widespread prevalence of this ailment is not without consequences, as alcohol use accounted for 2.44 million deaths in 2019 while being the leading risk factor for both burden of disease in individuals aged 25–49 years and death and disability among individuals aged 15–49 years [4]. Given the heavy burden alcohol misuse has on society, significant efforts into understanding and preventing development and persistence of AUD are warranted.

While many of the environmental risk factors for AUD are well-documented, both the underlying and resulting neurological patterns reflective of AUD are not as clear [5]. Functional Magnetic Resonance Imaging (fMRI) measuring Blood Oxygenation Level-Dependent (BOLD) signal is one neuroimaging technique that enables investigation into brain function associated with AUD. Task-based fMRI has been implemented to identify abnormal activation patterns in heavy drinkers when presented with external stimuli [6,7,8]. These investigations have consistently identified increased activation in mesocortical-limbic circuits consisting of key Default Mode Network (DMN), Salience Network (SN), and Limbic Network (LN) nodes when presented with alcohol or salient cues [8,9,10,11,12,13,14]. In addition, task-based fMRI revealed abnormal activation patterns in posterior DMN nodes of alcohol-dependent individuals during motor tasks. These abnormalities led to the belief that dysfunction in regions of the brain resulting from alcohol consumption may cause reorganization of brain functioning and even posterior DMN uptake of functions like motor planning and motor execution generally conducted by the anterior DMN [7,15,16].

More recently, resting-state fMRI (rsfMRI) has been used to evaluate functional connectivity (FC) differences between AUD patients and healthy controls. Resting-state FC analyses have revealed abnormal patterns in AUD patients’ functional brain networks, including the DMN and mesocortical-limbic circuit, among others [13,17,18,19,20]. The so-called triple network consisting of the DMN, SN, and Central Executive Network (CEN) has also become a topic of interest in rsfMRI analyses due to its involvement in decision-making and alterations in a variety of neuropsychiatric disorders [21]. Drinking tendencies were shown to relate to interactions within and between these three networks [22,23,24], further highlighting the complex interactions of different regions that present in AUD. Further FC-based approaches have employed graph theory to analyze the entire brain as a network, revealing further abnormal patterns in the triple network and across the brain [23,25,26,27,28].

Alternatives to connectivity analyses like Amplitude of Low Frequency Fluctuations (ALFF) and Regional Homogeneity (ReHo) have also been utilized in alcohol research to enable analysis of regional activity in the resting state without the need for task-based contrasts [29,30]. ALFF calculates the amplitude of frequency fluctuations within a specific frequency band associated with neural activity in fMRI (~0.009 to 0.08) and is often interpreted as an indication of the strength of spontaneous neural activity of a given region [31,32,33]. Findings in alcohol research vary, but significant differences in regional ALFF values between AUD patients and healthy controls have been consistently observed, often in many of the same key areas found in connectivity analyses such as the SN, LN, and DMN [34,35,36,37,38,39]. Notably, Song et al. showed that ALFF can be highly effective in classifying subjects as healthy control or AUD based on rsfMRI data alone [39]. ReHo also assesses the fMRI time series data but focuses on the similarity of BOLD signals for a neighborhood of voxels. Higher ReHo values for a neighborhood are believed to indicate higher levels of local communication and coherence, reflecting synchronized local activity [31,39,40,41,42]. Again, findings across studies indicate abnormal ReHo values in AUD patients, and ReHo proved to be a highly effective technique in classifying AUD based on rsfMRI signal [36,38,39,43].

One area of regional activity analysis yet to be explored in alcohol misuse is the fractal complexity of regional BOLD signal. Fractals are a natural phenomenon that present self-similarity across scales in space or time [44]. Both temporal and spatial fractals often manifest in physiology, whether through time series or anatomical structure [45,46,47,48,49]. These fractals present in many different physiologic systems, including brain activity [46,50,51,52,53]. The Hurst Exponent (HE), a measure of the fractal nature of a time series, has revealed abnormal fractal patterns in brain activity among a variety of neurological disorders [54,55,56,57,58,59]. Fractal complexity of structural and functional brain measures has been used in a few studies of substance use disorders, revealing unique fractal patterns associated with addiction [60,61,62]. Specifically, previous research identified abnormal fractal complexity of cortical folding in people that use drugs and individuals with poorer inhibitory function [60,62] as well as abnormal functional brain networks constructed using fractal dimension analysis in people that use drugs [61]. Despite its utility in deepening knowledge about the neural pathophysiology in disease and addiction, knowledge about the fractal complexity of brain activity in alcohol misuse is absent.

To broaden the knowledge of brain activity and its relationship with the development of alcohol misuse, we compared the fractal complexity of the BOLD signal measured using the HE in young adult risky drinkers to age-matched light drinkers. Additionally, we compared fractal complexity of BOLD signal between the same groups of participants at a younger age before developing any risky drinking habits. By comparing eventual risky drinkers to younger light drinkers who did not go on to exhibit risky drinking habits, we aimed to identify whether differences in brain activity patterns associated with alcohol consumption are present before any risky drinking behaviors develop. We employed ALFF and ReHo analysis alongside HE analysis as two methods that, while they have shown to be effective in characterizing AUD, are unexplored in younger adults regarding the development of unhealthy drinking habits. All three measures have exhibited strong stability/reliability in previous fMRI study [51,63], and each measure is understood to quantify different phenomena regarding brain activity, so employing all three measures provides deeper insight than a single method alone. Together, these three techniques serve to more fully elucidate abnormal brain activity patterns that characterize the development or risk of alcohol misuse.

## 2. Materials and Methods

### 2.1. Participants

All imaging and behavioral data was obtained from the National Consortium on Alcohol and NeuroDevelopment in Adolescence (NCANDA) [64]. The NCANDA study focuses on alcohol consumption and associated behavioral/demographic measures in adolescence and young adulthood, also acquiring rsfMRI images and diffusion tensor imaging (DTI) white matter structural scans. Data was acquired longitudinally for each individual every year after baseline visits, enabling investigation into drinking patterns and brain activity throughout development.

We conducted a group comparison between light drinkers and more moderate drinkers who exhibited risky drinking behaviors defined by the National Institute on Alcohol Abuse and Alcoholism (NIAAA) [65]. Specifically, we designated risky drinkers as individuals who exhibited regular (weekly) binge drinking behavior and drank on 100 or more days in the previous year [66]. The NIAAA defines binge drinking as consuming enough alcohol to raise blood alcohol concentration above 0.08% [66]. For male participants in this study, this generally corresponds to consuming five or more drinks within two hours, and about three to four drinks in the same timespan indicates binge drinking among female participants [65,66]. Though risky drinking behavior can be classified as even a single instance of binge-drinking as an adolescent, we implemented the additional threshold of 100 days of drinking in the previous year to ensure a pattern of alcohol consumption rather than individual instances or stretches of regular binge drinking behavior [67]. Among participants who exhibited multiple years of risky drinking behavior, data was selected from the year in which they drank most frequently. Light drinking criteria were defined as having drank on fewer than 20 days in the previous year and no regular episodes of binge drinking, despite being age 21 or older. Additionally, we only included participants who also exhibited non/light drinking behavior at a younger age, which enabled a group comparison between the same subjects before developing any risky drinking behaviors. With this form of case–control framework, we aimed to determine whether differences in brain activity patterns between light and risky drinkers were present before the development of risky drinking habits [68,69,70]. For each participant, the paired, younger data was selected as the earliest visit available in the NCANDA dataset at which the participant exhibited light/non-drinking behavior. To reiterate, the resulting four participant groups were risky drinkers, eventual risky drinkers, light drinkers, and younger light drinkers. Risky drinkers exhibited binge/risky drinking behavior, and eventual risky drinkers were the same individuals at earlier ages, before partaking in any risky drinking behavior. Light drinkers did not exhibit consistent binge/risky drinking behavior despite being of legal drinking age in the United States, and the younger light drinkers were again the same individuals as the light drinkers at earlier ages.

### 2.2. Data Acquisition and Preprocessing

NCANDA obtained longitudinal MRI data for each participant. Resting-state fMRI protocol was as follows: Repetition time (TR) of 2200 ms, echo time of 30 ms, 10 min scan length (274 volumes). Structural image acquisition protocol varied among scanners used at different data acquisition sites, but all imaging data was obtained using 3T scanners (GE Discovery MR750, GE HealthCare, Chicago, IL, USA) or (Siemens TIM TRIO, Siemens AG, Munich, Germany). For greater detail, refer to prior publications on NCANDA MRI acquisition [71].

We utilized Statistical Parameter Mapping 12 (SPM) and MATLAB R2023B for all preprocessing of MRI/fMRI data. Given the nature of the time-series analysis in this work, to limit any unintentional alterations to BOLD signal during preprocessing, all measures were calculated in each subject’s native space and then warped to standard space. Specifically, we conducted standard preprocessing steps in slice-time correction and realignment in native space [26]. Before any warping or coregistration, we applied a temporal bandpass filter (0.009–0.08 Hz) then regressed out six affine motion parameters, global mean cerebrospinal fluid signal, and global mean white-matter signal [72,73,74,75]. Motion scrubbing was also performed in native space, with volumes removed when framewise displacement exceeded 0.5 mm and mean percent signal change exceeded 0.5 (See group details in Table A1). We calculated all values of interest (HE, ALFF, ReHo) for each voxel (4 × 4 × 5 mm^3^) in native space, saving brain maps for each measure. Each brain map was then coregistered alongside the functional data to the structural image, after which they were warped into standard Montreal Neurological Institute (MNI) space and resliced to 3 × 3 × 3 mm^3^ to match structural data for group comparisons.

### 2.3. Measure Calculations

We investigated the three unique measures of BOLD signal activity using MATLAB R2023B. There are a multitude of ways to estimate fractal complexity through HE, so we elected to use two techniques shown in previous work to be most effective in estimating HE in BOLD signal within the frequency band enforced by temporal filtering [72,76]: the Generalized Hurst Exponent (GHE) and Higuchi Fractal Dimension (HFD) [77,78]. These methods outperformed alternative Detrended Fluctuation Analysis (DFA) and wavelet-based estimator methods on bandpass-filtered data, and they were generally more effective in revealing activation differences across contrast [76]. The generalized hurst exponent follows the below equation [77]:

$$S\left(q,\tau \right)=\left\langle {\left|x\left(t+\tau \right)-x(t)\right|}^{q}\right\rangle \propto \tau^{qGHE\left(q,\tau \right)},$$

where *x*(*t*) indicates the BOLD signal at time *t*, *q* represents the ordered moment of the distribution of increments. *τ* represents a varied time lag and *S* represents change in signal as a factor of change in time, which enables a fit between the log of *τ* values and log of *S* values. The mean slope of this fit across different *τ* values is then extracted as the *GHE*. We implemented this process using the genhurst function available through the MATLAB file exchange [79] with a max *τ* value set at 10 and *q* = 2 [76]. With a limited scan duration of 10 min and TR of 2.2 s, a shorter *τ* value than the default was necessary to retain linearity in the above equation. The second order GHE (*q* = 2) was selected due to slightly increased performance in the aforementioned study evaluating different estimations of HE [76].

The Higuchi Fractal Dimension follows a complex process, where we first define new time series [78],

$$x_{\tau }^{t}:x\left(t\right),\;x\left(t+\tau \right),\;x\left(t+2\tau \right),\ldots ,\;x\left(t+\left[\frac{N-t}{\tau }\right]\tau \right),\;\left(t=1:\tau \right),$$

where *x*(*t*) again indicates the BOLD signal at time *t*, *τ* indicates a consistent time lag, *N* is the length of the BOLD signal, and [] indicates Gauss’ notation. The length of the curve, $L_{t}(\tau )$, of each series can then be defined as follows [78]:

$$L_{t}\left(\tau \right)=\;\left\{\left(\sum_{i=1}^{\left[\frac{N-t}{\tau }\right]}\left|x\left(t+i\tau \right)-x(t+(i-1)\tau \right|\right)\frac{N-1}{\left[\frac{N-t}{\tau }\right]\tau }\;\right\}/\tau .$$

Finally, the fractal dimension is defined using the following relationship [78]:

$$\langle L(k)\rangle \propto \tau^{-D},$$

where 〈*L*(*k*)〉 denotes the average length of the curve value across all values *τ*. The Hurst exponent is then estimated as 2 − *D*. For implementation in MATLAB, we utilized the hfd command available through the MATLAB file exchange [80] with *τ* = 5 samples [76]. Again, we selected a lower *τ* value of 5 due to the shorter scan duration and TR value slightly greater than 2.0 s.

To calculate *ALFF* values, we used MATLAB’s Fast Fourier Transform (*FFT*) command to generate the frequency spectra for each time series, after which we calculated the mean of the inverse square of absolute magnitude at each frequency within the aforementioned bandpass filter [29].

$$ALFF=\frac{1}{0.08-0.009}\int_{0.009}^{0.08}\sqrt{\left|FFT(x)\right|},$$

where *FFT*(*x*) indicates the Fast Fourier Transform of the BOLD signal at a given voxel.

To generate ReHo values for each voxel, we employed the y_reho function within the Data Processing Assistant for Resting-State fMRI (DPARSF) toolbox [30,81]. ReHo calculates Kendall’s coefficient of concordance (*KCC*) for a given voxel and its 26 neighbors [82], via the following equation [30]:

$$KCC=\;\sum 12\times \left[(R_{t}{)}^{2}-N(R{)}^{2}\right]/K^{2}/(N^{3}-N).$$

Here, $R_{t}$ indicates the sum rank at time point *t*, *N* denotes the total number of time points, *R* represents the mean across all $R_{t}$ values, and *K* indicates the number of voxels within a neighborhood. We employed a *K* value of 27, as is standard [81,83,84].

### 2.4. Group Comparisons

**Figure 1:**
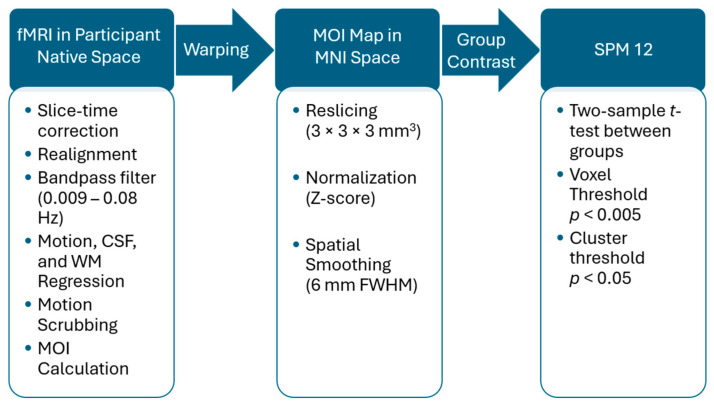
Workflow detailing the generation of contrast maps comparing measures of interest (MOI) between groups. All steps were completed in MATLAB R2023B and Statistical Parameter Mapping (SPM 12). Processing steps are shown in order of completion, starting in participant native space and proceeding down followed by right.

HE, ALFF, and ReHo values at each voxel in the brain were compared between risky and light drinkers as well as between the eventual risky and younger light groups at earlier points, before exhibiting any risky drinking behaviors. We normalized values into z-scores for each participant to account for global differences, then applied a 6 × 6 × 6 mm FWHM gaussian spatial smoothing filter [32,85]. Two-sample *t*-tests were then conducted using SPM to compare values of interest of both groups at each voxel in standard space [32]. We implemented a voxel-wise *p*-value threshold of 0.005 (*t* statistic > 2.632, Cohen’s d > 0.557) and defined significant clusters of voxels using cluster-corrected *p*-values at or below 0.05 [86,87,88]. As each measure results in a different level of perceived spatial smoothing, the minimum cluster size differed between measures. Minimum cluster sizes for each measure were calculated as follows: GHE—26 voxels, HFD—24 voxels, ALFF—24 voxels, ReHo—29 voxels. See the full workflow from preprocessing to contrast maps in Figure 1.

To aid with interpretation, we also generated normalized group-mean brain maps for each value of interest. These maps served to visualize group differences and regions whose values of interest are generally elevated or reduced.

## 3. Results

### 3.1. Groups

The groups were compared based on a multitude of demographic, imaging, and behavioral variables (Table 1). The risky drinker group consisted of 38 individuals (15 females) while the lighter drinker group consisted of 54 individuals (26 females). The eventual risky drinkers consisted of the same cohort of risky drinkers at earlier ages, before developing any risky drinking habits. Similarly, the younger, light drinkers consisted of the light drinkers at younger ages to compare with the eventual risky drinkers. Both pairs of groups were not significantly different in age, sex proportion, or MRI scanner proportions (GE MR 750 vs. Siemens TrioTrim), but both exhibited a significantly different number of days drank and binge drinking occasions in the prior year. In the case of the younger group, however, both groups exhibited very light drinking patterns and minimal binge drinking. We also compared the groups in the number of other drugs used and responses to key questions from the UPPS-P Impulsive Behavior examination to discern differences in response to exciting stimuli and impulsivity [89,90]. The risky drinking group, as expected, exhibited increased substance use overall with the increased alcohol consumption and use of other drugs. The UPPS-P 31 measures a participant’s affinity for new and exciting sensations, and the UPPS-P 46 measures a participant’s particular affinity for the exciting sensation of skiing down a mountain, where lower numbers indicate greater affinity in both cases. The risky drinking group exhibited slightly decreased values for both surveys compared to the light drinking group, though differences were not significant. Eventual risky drinkers also had decreased values compared to the younger light drinkers, with only the UPPS-P 46 difference significant.

### 3.2. Group-Average Maps

**Figure 2:**
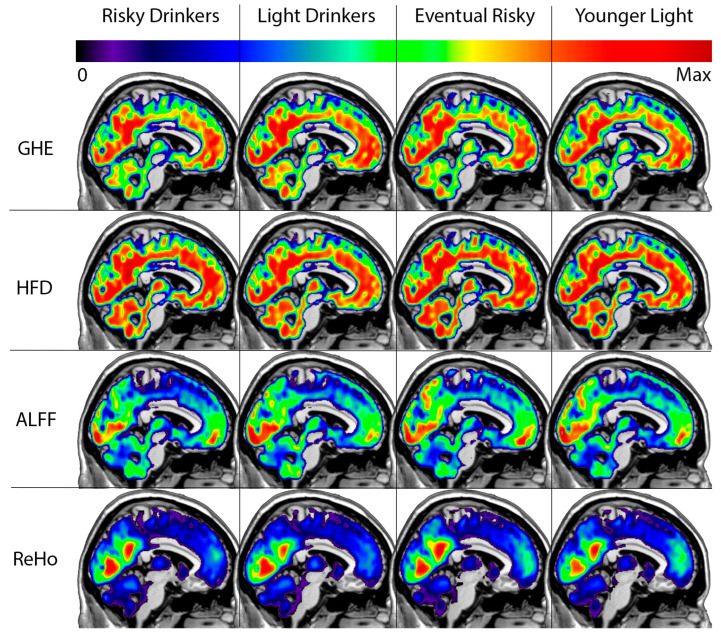
Group-average, normalized value of interest maps for the Generalized Hurst Exponent (GHE), Higuchi Fractal Dimension (HFD), Amplitude of Low Frequency Fluctuations (ALFF), and Regional Homogeneity (ReHo). Only positive normalized (Z-score) values are shown for better contrast in regions of interest. Sagittal slices are shown at MNI X coordinate −6. Cooler colors indicate low values in the respective measure of interest, while hotter colors indicate high values in the respective measure of interest relative to remaining cortical areas. Given the varying dispersity of values, the particular color scale for each measure ranged from zero to the maximum z-score for each measure.

With normalized value of interest maps for each participant within the four groups, the mean value within each group was calculated for each voxel. Full-brain average maps were thus generated for each analytical technique and each group (Figure 2). Strictly positive values are shown to highlight contrast between cortical areas of interest. In doing so, we aimed to identify areas of relatively high and low HE, ALFF, and ReHo values to aid with interpretation and validate measure calculations by comparing with expectations based on literature [91,92,93,94,95]. We also calculated the spatial correlation between group mean maps of different measures, and the mean correlation between measures was calculated from these values across all groups.

As expected, GHE and HFD maps were similar (r = 0.9984), a positive indicator that they are similarly equipped for measuring fractal complexity. The prefrontal cortex and precuneus stand out as generally high values across all groups. Compared to ALFF and ReHo maps, high HE values are much more distributed across the brain. ALFF maps tend to show the greatest values concentrated in occipital and inferior prefrontal areas and tend to be decreased in the cerebellum compared to HFD and GHE maps. Still, ALFF maps were somewhat similar to GHE maps (r = 0.9577) and HFD maps (r = 0.9532). ReHo is generally similarly high in the occipital and precuneus areas, with notably low values in the inferior prefrontal cortex that is a stark contrast to the remaining measure maps. ReHo maps were generally more distinct compared to GHE maps (r = 0.8427), HFD maps (r = 0.8350) and ALFF maps (r = 0.8277).

### 3.3. Risky vs. Light Drinkers

**Figure 3:**
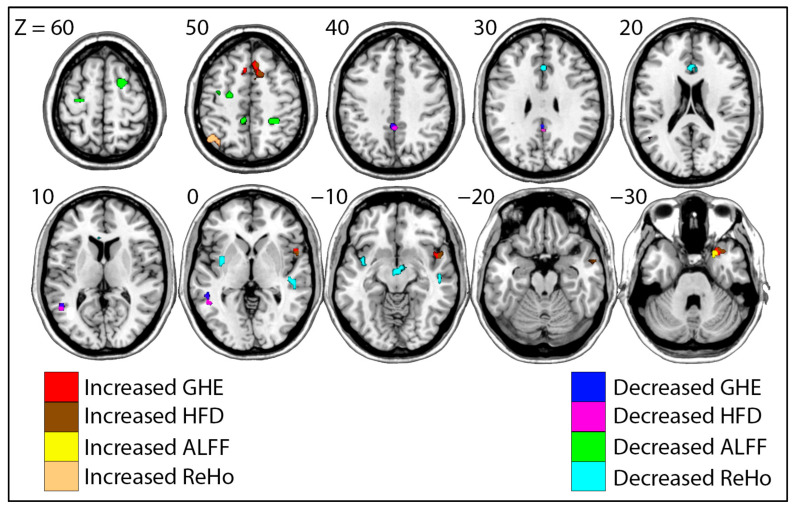
Risky drinkers vs. age-matched light drinkers. Significant clusters of different values for Generalized Hurst Exponent (GHE), Higuchi Fractal Dimension (HFD), Amplitude of Low Frequency Fluctuations (ALFF), and Regional Homogeneity (ReHo) are shown. Warmer colors indicate values that were greater in risky drinkers (GHE = Red, HFD = Brown, ALFF = Yellow, ReHo = Orange), while cooler colors indicate values that were lower in risky drinkers (GHE = Blue, HFD = Pink, ALFF = Green, ReHo = Cyan). MNI Z coordinates for each slice are shown.

Two-sample *t*-tests were conducted between the groups of risky drinkers and light drinkers for each value of interest. Significantly different clusters of voxels were identified using SPM, as shown in Figure 3 and Table 2. Anatomical locations of each cluster were identified using the Automated Anatomical Labelling Atlas 3 (AAL3) [96].

We observed multiple differences between the risky and light drinkers for each measure of interest. Nearly identical clusters were identified via GHE and HFD, with risky drinkers exhibiting increases in the prefrontal cortex and right anterior insula as well as decreases in the temporal and parietal lobes. ALFF values were also increased in risky drinkers in the inferior prefrontal cortex, but they were significantly decreased in portions of the parietal and superior prefrontal lobes. Risky drinkers showed increased ReHo values in the parietal lobe, with significant decreases in the midbrain, temporal lobe, prefrontal lobe, and left insula.

### 3.4. Early Comparison

**Figure 4:**
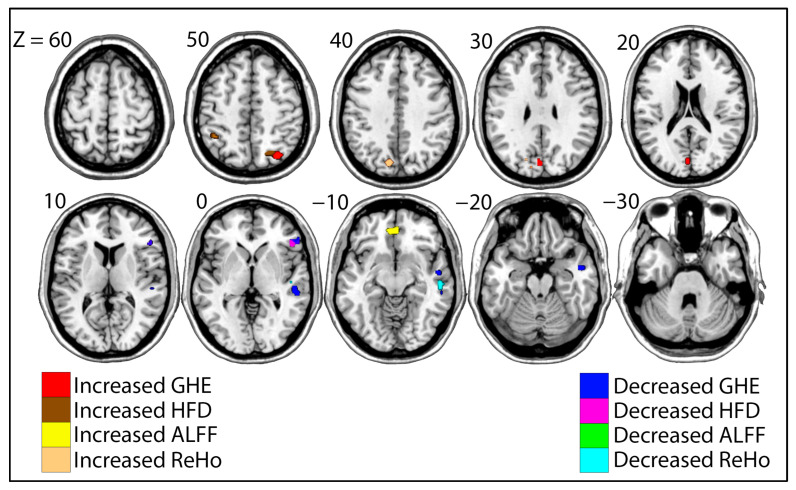
Eventual risky drinkers vs. younger light drinkers. Significant clusters of different values for Generalized Hurst Exponent (GHE), Higuchi Fractal Dimension (HFD), Amplitude of Low Frequency Fluctuations (ALFF), and Regional Homogeneity (ReHo) are shown. Warmer colors indicate values that were generally greater in eventual risky drinkers (GHE = Red, HFD = Brown, ALFF = Yellow, ReHo = Orange), while cooler colors indicate values that were generally decreased in eventual risky drinkers (GHE = Blue, HFD = Pink, ALFF = Green, ReHo = Cyan). MNI Z coordinates for each slice are shown.

We also conducted two-sample *t*-tests between the groups of eventual risky drinkers and younger light drinkers for each value of interest to clarify whether differences between risky and light drinkers are present prior to risky drinking habits. Significant regional differences are shown in Table 3 and Figure 4.

In comparing the eventual risky drinkers to those who did not proceed to exhibit risky drinking behavior, eventual risky drinkers showed increased GHE and HFD values in the right superior parietal lobe and decreased values in the right inferior frontal lobe. The eventual risky group also showed increased GHE in the left occipital lobe and decreased GHE in the right superior temporal lobe, while increases in HFD also presented in the left inferior parietal lobe. Increases in ALFF values in the eventual risky drinking group were observed in the inferior prefrontal lobe. We also identified increases in eventual risky drinkers’ ReHo values in the left occipital lobe and decreases in the right superior temporal lobe.

## 4. Discussion

We observed significant differences in each measure of interest between risky/light drinkers and between the same participant groups (eventual risky/younger light) years earlier—before any risky drinking habits had developed. Within the older groups, we identified significantly increased GHE and HFD values in the risky drinkers in the right orbitofrontal cortex (OFC), right anterior insula, and medial superior prefrontal cortex; we identified decreased values in the left middle temporal gyrus and medial precuneus/posterior cingulate cortex (PCC). These increased HE values indicate longer memory or self-referential BOLD time series, while decreased HE values may show more erratic or dynamically evolving BOLD signal. Previous research on the HE has shown that values decrease during complex cognitive tasks [50,51,53,91,97,98]. HE is not directly related to activation, however. While many networks exhibit decreased HE values during task-induced activation, the DMN shows high HE values during rest, as exhibited by the group average maps generated in this work [91]. Involved in self-referential thought, the DMN is minimally concerned with outside influences at rest [99]. Consequently, the widespread contention is that HE decreases based on attention and processing of outside information [50]. Thus, in the resting-state analysis here, more risky drinkers may exhibit decreased external processing and increased self-referential thought in key anterior DMN and SN nodes that are highly involved in processing and making decisions regarding salient stimuli [100,101,102,103]. Such a case would potentially indicate a desensitization or compensatory mechanism in which limited attention is paid to external stimuli when alcohol influences are absent. Previous research has demonstrated that AUD patients may exhibit attentional bias toward salient, alcohol-related stimuli and away from other stimuli [104]. UPPS-P responses may support this phenomenon, with the risky drinking group slightly more welcoming to exciting stimuli compared to the light drinking group. Additionally, the risky drinking group responses changed towards being less affected by exciting stimuli compared to their younger, non-drinking selves, while the light drinkers were generally more or similarly welcoming of new and exciting stimuli compared to their younger, non-drinking selves. While changes were not significant, these trends support the premise that repeated alcohol use may shift perception of stimuli to highlight substance or exciting stimuli. Thus, it is possible that in the resting-state with no salient/alcohol-related stimuli present, these nodes pay less attention to outside stimuli in risky drinkers than their counterparts in the light drinkers. In contrast, two posterior nodes of the DMN exhibit decreased HE values in the risky drinking group. This observation aligns with previous theories that aberrations to the anterior DMN result in extended effects to the posterior DMN [9,19]. Specifically, the PCC in AUD patients has shown increased activation during complex cognitive tasks, leading to the belief that a compensatory mechanism exists in which parietal DMN regions become increasingly involved with complex tasks and less involved in self-referential thought [105,106,107]. Such involvement would be consistent with the decreased HE values observed here.

In the same groups, we showed significantly increased ALFF values in the risky drinkers in the right orbitofrontal cortex and decreased ALFF values in the right postcentral, right superior frontal lobe, left precentral, and precuneus. ALFF values are believed to quantify strength of spontaneous neural activity, and they increase with regional activation, as evidenced by high ALFF values in DMN regions shown in group average maps [31,32,33,92]. Consequently, the two significant overlapping clusters in the precuneus and orbitofrontal cortex can be indicative of the same phenomena observed through the HE analysis. At rest, increased activation in the DMN is indicative of self-referential thought, so increased ALFF in the OFC may therefore suggest increased self-referential thought and decreased processing of external stimuli. Notably, this postulation aligns with the observed HE increase in the OFC, and the decreased ALFF in the PCC indicates less engagement in self-referential thought also reflected by decreased HE. On the other hand, the other regions of decreased ALFF in risky drinkers require further research. These three clusters overlap with portions of the SensoriMotor Network (SMN), a network primarily involved in motor and sensory function [108,109]. Decreased ALFF values in risky drinkers could indicate decreased neural activity in these SMN regions at rest. Previous research has identified motor dysfunction in AUD, with abnormal activation in parietal SMN regions during motor tasks in AUD [7,15]. Given the interaction between the SMN and insula related to perceiving and weighing stimuli, the dysfunction recognized through previous research and decreased ALFF here could coincide with decreased neural activity in these SMN regions [9,102,103].

Significant differences between the risky and light drinkers were also observed in ReHo values, with a significantly increased cluster in risky drinkers in the left inferior parietal lobe and significantly decreased clusters in the midbrain, right superior temporal lobe, ventromedial anterior cingulate cortex, and left insula. ReHo represents the similarity in BOLD signal in local voxels, generally indicating local coherence and communication, thus high values in large visual areas are shown through the group average maps likely due to the strong local synchronization in the visual cortex that occurs during an unchanged visual stimulus at rest [39,40,41,42,93,94,95]. Significant decreases in ReHo in three key nodes of the SN may indicate decreased local coherence in these regions as well as a portion of the midbrain. Both the midbrain and SN exhibit abnormalities in AUD, and they are both associated with processing pre-perceived stimuli [102,110]. The potential attentional bias where heavier drinkers exhibit altered perception of stimuli based on its association with alcohol may present alongside disrupted local coherence/communication in these nodes that play key roles in placing value on perceived stimuli [100,101,104]. The left inferior parietal lobe, however, showed increased ReHo in risky drinkers, suggesting this region showed increased local coherence in the risky drinkers. This phenomenon could be a corollary of the decreased parietal and superior prefrontal ALFF activity. A multi-network hub also associated with attention and perception of stimuli, increased ReHo in this region may also be indicative of decreases in this specific function [111,112]. Essentially, decreased activity directed towards attention/stimuli perception enables more activity towards internal thought/reflection, leading to greater BOLD correlation with the neighboring (and potentially overlapping) voxels of the DMN’s lateral parietal node [99,113].

**Figure A1:**
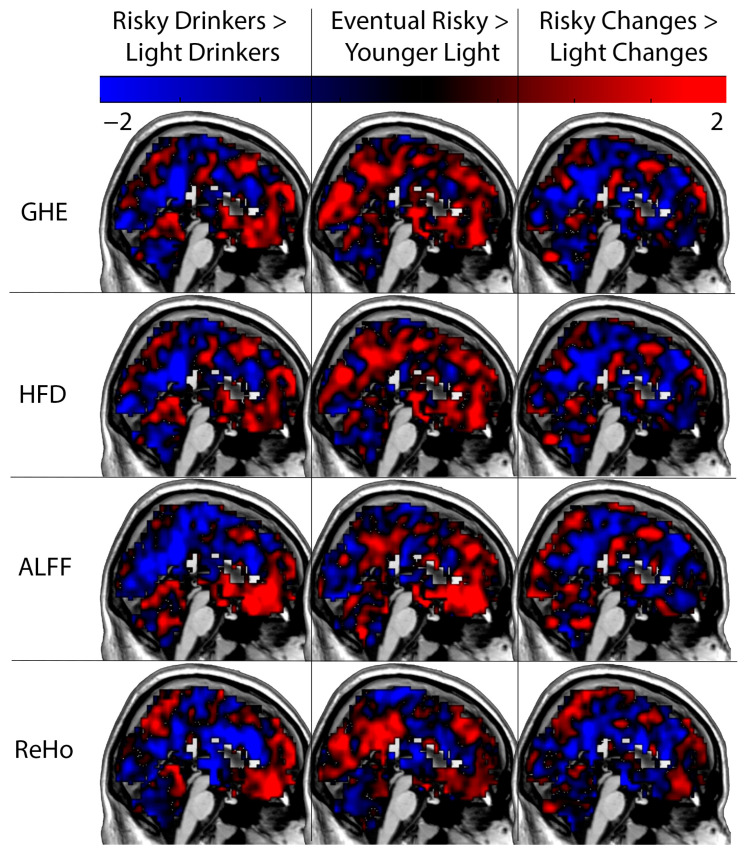
T value contrast maps for Generalized Hurst Exponent (GHE), Higuchi Fractal Dimension (HFD), Amplitude of Low Frequency Fluctuations (ALFF), and Regional Homogeneity (ReHo) values between groups of risky drinkers and light drinkers as well as their younger, light drinking selves. We also calculated the differences from younger to older visits for each participant, contrasting the eventual risky to risky drinkers changes with the younger light to light drinkers changes. Significant differences within the maps are detailed in the main text. Red colors indicate increased values in the risky groups. Sagittal maps shown at MNI X coordinate 1.

Identical analyses were conducted on groups of the same individuals at younger, light-drinking states to provide information on whether the above differences are indicators of likelihood to develop drinking habits (risk factors) or effects of drinking habits. Importantly, the patterns that significantly differentiated risky and light drinkers were not present when both groups were younger, non/light drinkers, providing some evidence that these differences may result from drinking patterns rather than causing them. Furthering this point, the general contrast maps show notably different patterns between the two pairs of groups for each measure (Figure A1). Thus, the differences in brain activity pattens between the older groups were largely dissimilar from the differences between the younger groups. Admittedly, key similarities in the contrast maps present in the inferior prefrontal cortex, a region consistently identified as abnormal in previous research and in this work [13,18,27,34].

The significant differences between the younger groups, while distinct from differences revealed between the older groups, may be indicative of potential risk factors for the development of risky drinking habits. ALFF values were significantly increased in the inferior prefrontal cortex in eventual risky drinkers, pointing to the possibility of increased activation in the resting-state, consistent with greater self-referential thought. HE values were also significantly increased in this group in the parietal cortex, which may indicate decreased capacity for external processing [50]. Both these regions are thought to be involved in inhibition, potentially indicating decreased inhibition in the group of participants who would go on to develop risky drinking habits [114,115,116,117]. Additionally, we identified significant HE decreases in the eventual risky drinking group in portions of the SN, possibly representing greater attention placed on external stimuli. While this trend is opposite the findings of the older group, it is reasonable to theorize that the eventual risky drinkers may be more influenced by salient stimuli than the strictly light drinking group before attentional bias develops as a result of increased alcohol consumption [104]. Given the eventual risky group’s increased affinity for exciting stimuli exhibited by UPPS-P 46 as well as the decrease in the risky group’s affinity during the older visits, this hypothesis seems even more likely. This phenomenon combined with the potentially decreased inhibition could certainly explain the group’s inclination towards more risky drinking patterns. Both decreased inhibitory control and increased inclination to stimuli in the form of substance have shown associations with likelihood to develop drinking/drug habits [118,119,120]. Nonetheless, these conclusions are conjecture based on the findings herein, and further research should be conducted to validate these potential phenomena.

Importantly, the two pairs of groups were not significantly different in age or sex proportions, and both groups had similar proportions of participants between the two different MRI scanners. All three measures are known to change with age [73,97,121,122] and differ based on sex or MRI scanner [76,122,123,124], so identifying groups without significant differences in these variables increases the likelihood that discovered differences are truly associated with alcohol consumption. The risky drinking group consumed significantly more alcoholic beverages than the light drinking group, as was intended with group selection. As stated, identifying and classifying neurological risk factors and effects of drinking in younger individuals before the development of AUD is critical to prevention, but it does mean that effect sizes may be smaller than analyses on AUD patients and healthy controls. Though cluster correction is an effective method at identifying significantly different clusters of activity in the brain, the limited effect size prevents severely low thresholds. Thus, false positive findings are possible, and any findings herein should be validated in future research. Additionally, it should be mentioned that the risky and light groups did differ in patterns of drug use, with the risky group exhibiting increased drug use compared to the light group. While this may slightly limit strength of interpretation, there is certainly overlap between both risk factors and effects of alcohol and drug use, thus the results herein may be valuable for understanding neurological effects and risk factors underlying substance use [125,126,127]. In general, this work is largely exploratory, and findings should be replicated in future research before being widely accepted.

As is the case with most alcohol research, this work is limited in its capacity to capture all the factors that contribute to alcohol use. Environmental factors play a large role in developing drinking habits, and gathering the full context of how an individual’s environment interacts with their brain is not possible [120,128]. Furthermore, though the NCANDA study is a paramount step in furthering longitudinal alcohol research, information on participant drinking habits is limited to the ten years since it began [64]. Thus, longer-term drinking patterns for the participants within are unknown, limiting potential conclusions about risk factors for unhealthy drinking patterns. Additionally, though the NCANDA study did employ exclusion criteria limiting potential confounding effects like psychiatric disorder and certain medication use, other factors contributing to the measures employed herein may be unaccounted for. Again, continued work in evaluating both neuroimaging and environmental factors that may indicate a predisposition to alcohol use are critical.

## 5. Conclusions

We evaluated regional fMRI-measured BOLD series using HE, ALFF, and ReHo in a cohort of young adults and adolescents who exhibited varying drinking behaviors to bolster the growing knowledge of the relationship between alcohol consumption and the developing brain. Significant differences in regional activity patterns presented between risky drinkers and light drinkers, many of which aligned with previous research exposing abnormal functioning of regions involved in reward and inhibition pathways. We also identified significant differences in the groups of individuals at younger ages, before any risky drinking habits had developed, which differed from those found after risky drinking habits had developed. Though theories formed on these findings align with behavioral findings in scientific literature, they warrant further validating research. We provided novel information regarding the fractal nature of brain activity patterns in different drinking patterns while adding to existing literature on ALFF and ReHo in different levels of alcohol consumption.

## Figures and Tables

**Table 1:** Demographic and drinking information of each group. *p*-values were calculated using two-sample *t*-tests between the groups. The first row shows the count of females in each group as well as the percentage of members of said group that were female, and the second row shows the number and percentage of individuals whose data was obtained using the GE MR 750 MRI scanner. The third, fourth, and fifth row show mean and standard deviation (parenthesized) of age, number of days drank in the previous year, and number of binge drinking episodes in the previous year, respectively, for each group. We also calculated mean and standard deviation of other drugs used as well as responses to the UPPS-P Impulsive Behavior examination, where lower numbers indicate affinity for new and exciting sensations.

Group	Risky	Light	*p*-Value	Eventual Risky	Younger Light	*p*-Value
**Females**	15 (39%)	26 (48%)	0.41	15 (39%)	26 (48%)	0.41
**GE MR 750 Scanner Count**	21 (55%)	33 (61%)	0.57	21 (55%)	33 (61%)	0.57
**Age**	21.04 (1.50)	21.37 (0.28)	0.19	17.01 (1.52)	17.23 (0.78)	0.42
**Days Drank in Previous Year**	150.92 (45.03)	6.20 (5.25)	<0.001	3.08 (4.4)	0.85 (2.33)	0.006
**Binge Drinking Occasions in Previous Year**	78.66 (42.77)	0.70 (1.41)	<0.001	1.05 (2.29)	0.00 (0.0)	0.001
**Other Drugs Used**	2.32 (2.57)	0.20 (0.63)	<0.001	0.00 (0.0)	0.02 (0.14)	0.4
**USSP-P 31**	1.81 (0.79)	1.85 (0.77)	0.81	1.73 (0.61)	1.96 (0.87)	0.14
**USSP-P 46**	2.14 (1.07)	2.55 (1.12)	0.08	1.89 (0.91)	2.50 (1.08)	0.004

**Table 2:** Significantly different clusters of normalized Generalized Hurst Exponent, Higuchi Fractal Dimension, Amplitude of Low Frequency Fluctuations, and Regional Homogeneity values between risky and light drinkers. Positive T values indicate greater values in risky drinkers. Regions were defined using the Automated Anatomical Labelling Atlas 3 [96]. MNI coordinates convey the location of the peak *t* statistic within each cluster.

Region	Peak T	Voxels	*p*-Value	MNI Coordinates
*Generalized Hurst Exponent*				
R OrbitoFrontal Cortex	4.60	40	0.017	24 15 −24
R Anterior Insula	3.81	31	0.032	48 12 −3
Superior Frontal	3.60	42	0.015	12 15 51
L Middle Temporal	−3.50	37	0.021	−48 −54 15
Precuneus	−3.40	34	0.026	−3 −42 42
*Higuchi Fractal Dimension*				
R OrbitoFrontal Cortex	4.29	32	0.025	27 18 −24
Superior Frontal	4.06	41	0.013	9 18 54
R Anterior Insula	4.04	51	0.007	48 9 −6
L Middle Temporal	−3.65	38	0.016	−48 −54 15
Precuneus	−3.47	33	0.023	0 −45 39
*Amplitude of Low Frequency Fluctuations*				
R OrbitoFrontal Cortex	4.41	40	0.013	27 18 −24
R Postcentral	−4.01	27	0.036	27 −39 48
L Precentral	−3.73	47	0.008	−33 −9 57
R Superior Frontal	−3.70	25	0.042	18 6 60
Precuneus	−3.58	41	0.012	−6 −42 48
*Regional Homogeneity*				
L Inferior Parietal	3.68	35	0.033	−48 −63 51
Midbrain	−4.53	30	0.047	9 −6 −12
R Superior Temporal	−4.07	60	0.008	48 −24 −3
Ventromedial Anterior Cingulate Cortex	−3.79	36	0.031	3 27 33
L Insula	−3.29	29	0.050	−39 3 −9

**Table 3:** Significantly different clusters of normalized Generalized Hurst Exponent, Higuchi Fractal Dimension, Amplitude of Low Frequency Fluctuations, and Regional Homogeneity values between eventual risky and younger light groups. Positive T values indicate greater values in the group of individuals who would go on to exhibit risky drinking behavior. Regions were identified using the Automated Anatomical Labelling Atlas 3 [96]. MNI coordinates convey the location of the peak *t* statistic within each cluster.

Region	Peak T	Voxels	*p*-Value	MNI Coordinates
*Generalized Hurst Exponent*				
R Superior Parietal	4.02	32	0.028	33 −69 51
L Cuneus	3.75	29	0.035	0 −81 27
R Superior Temporal	−3.64	38	0.018	54 −27 0
R Superior Temporal	−3.36	34	0.024	54 −6 −12
R Inferior Frontal	−3.35	30	0.033	51 27 12
*Higuchi Fractal Dimension*				
R Superior Parietal	3.95	43	0.010	33 −69 51
L Inferior Parietal	3.66	27	0.035	−33 −51 45
R Inferior Frontal	−3.58	28	0.032	51 27 12
*Amplitude of Low Frequency Fluctuations*				
Inferior Frontal (OFC)	3.89	47	0.006	−6 45 −12
*Regional Homogeneity*				
L Cuneus	4.10	30	0.046	−12 −75 39
R Superior Temporal	−3.42	30	0.046	57 −24 −6

## Data Availability

The data presented in this study are openly available in NCANDA at https://ncanda.org/datasharing.php (accessed on 1 August 2025). MATLAB commands are available through the MATLAB file exchange, and any specific scripts used can be made available upon request to the corresponding author.

## Abbreviations

The following abbreviations are used in this manuscript:

AUD	Alcohol Use Disorder
fMRI	Functional Magnetic Resonance Imaging
BOLD	Blood Oxygenation Level-Dependent
DMN	Default Mode Network
SN	Salience Network
LN	Limbic Network
rsfMRI	Resting State Functional Magnetic Resonance Imaging
FC	Functional Connectivity
CEN	Central Executive Network
ALFF	Amplitude of Low Frequency Fluctuations
ReHo	Regional Homogeneity
HE	Hurst Exponent
NCANDA	National Consortium on Alcohol and NeuroDevelopment in Adolescence
DTI	Diffusion Tensor Imaging
NIAAA	National Institute on Alcohol Abuse and Alcoholism
SPM	Statistical Parameter Mapping
MNI	Montreal Neurological Institute
GHE	Generalize Hurst Exponent
HFD	Higuchi Fractal Dimension
KCC	Kendall’s Coefficient of Concordance
FWHM	Full Width at Half Maximum
OFC	OrbitoFrontal Cortex
PCC	Parietal Cingulate Cortex
SMN	SensoriMotor Network

## Appendix A

We have also included data on contrasts between the changes in measures of interest with age among those who develop risky drinking habits and those who remain light drinkers. By calculating difference maps between younger and older visits for each participant, we conducted a two-sample *t*-test comparing the difference maps between the two groups. Significantly different clusters are shown in Table A3 and contrast maps are shown in Figure A1.

**Table A1:** Motion Scrubbing data for each group. Volumes were removed if framewise displacement exceeded 0.5 mm and mean percent signal change exceeded 0.5.

Group	Risky	Light	Eventual Risky	Younger Light
**Volumes Removed**	43	84	34	85
**Mean Framewise Displacement**	0.14	0.16	0.14	0.15

**Table A2:** Full drinking pattern distribution data for each group, where Q1 and Q3 indicate the first and third quartiles, respectively.

Group	Risky	Light	Eventual Risky	Younger Light
*Days Drank in Previous Year*				
Minimum	102.0	0.0	0.0	0.0
Q1	111.0	2.0	0.0	0.0
Median	140.0	5.0	0.0	0.0
Q3	182.0	9.0	1.0	0.0
Maximum	254.0	18.0	8.0	0.0
*Binge Drinking Occasions in Previous Year*				
Minimum	6.0	0.0	0.0	0.0
Q1	50.0	0.0	0.0	0.0
Median	80.5	0.0	0.0	0.0
Q3	102.0	1.0	1.0	0.0
Maximum	170.0	8.0	8.0	0.0

**Table A3:** Significantly different clusters of normalized Generalized Hurst Exponent, Higuchi Fractal Dimension, and Regional Homogeneity differences from eventual risky to risky and younger light to light. There were no significant differences exhibited through Amplitude of Low Frequency Fluctuations analysis. Positive T values indicate more positive changes in the group of individuals who would go on to exhibit risky drinking behavior compared to those who would not develop risky drinking behavior. Negative T values indicate more negative changes in the group of individuals who would go on to exhibit risky drinking behavior compared to those who would not develop risky drinking behavior. Regions were identified using the Automated Anatomical Labelling Atlas 3 [96].

Region	Peak T	Voxels	*p*-Value	MNI Coordinates
*Generalized Hurst Exponent*				
R Middle Temporal	3.85	42	0.014	54 −6 −2
L Inferior Temporal	3.56	26	0.046	−51 −48 −21
*Higuchi Fractal Dimension*				
R Superior Temporal	4.11	32	0.025	66 −42 21
R Inferior Frontal	4.02	26	0.040	48 27 15
L Putamen	3.59	25	0.044	−27 −15 3
R Middle Temporal	3.43	40	0.014	54 0 −21
R Precentral	−4.66	27	0.037	42 −18 42
*Regional Homogeneity*				
R Angular	3.69	30	0.047	27 −60 48
